# Poor prenatal service utilization and pregnancy outcome in a tertiary health facility in Southwest Nigeria

**DOI:** 10.11604/pamj.2020.35.28.20426

**Published:** 2020-02-06

**Authors:** Jacob Olumuyiwa Awoleke, Babatunde Ajayi Olofinbiyi

**Affiliations:** 1 Department of Obstetrics and Gynaecology, Ekiti State University, Ado-Ekiti, Nigeria

**Keywords:** Prenatal care, pregnancy outcome, low birth weight, universal coverage, Nigeria

## Abstract

**Introduction:**

Poor prenatal service utilization is common in developing countries. However, the predictors and pregnancy outcomes of poor utilizers have not been fully examined in our setting.

**Methods:**

Poor and good prenatal service utilizers were compared with respect to demographic characteristics and pregnancy outcomes in Ado-Ekiti, Nigeria.

**Results:**

Poor utilizers were significantly more likely to be single mothers, with unemployed husbands/partners, but less likely to have labour induction compared with good utilizers. Also, the women with fewer than four antenatal visits had significantly more babies with low birth weight (18% versus 9.8%, p = 0.003), and 5-minute Apgar scores less than 7 (17.9% versus 10.1%, p = 0.023). Multivariate regression analysis revealed that having an unemployed husband/partner (adjusted odds ratio (AOR): 2.33; 95% Confidence Interval (C.I.): 1.24 - 4.38; p = 0.009), with low birth weight babies (AOR: 1.66; 95% C.I.: 1.01 - 2.73; p = 0.045), and delivering without induction of labour (AOR: 4.27; 95% C.I.: 2.38 - 7.64; p < 0.001) were independently associated with poor prenatal service utilization.

**Conclusion:**

Efforts devoted to identifying women who are likely to be non- and poor-utilizers of prenatal care are recommended. Scaling up awareness campaigns on maximizing the benefits of prenatal care, increasing the content quality of antenatal visits to give women a positive pregnancy experience and implementing a National Health Insurance package that strategically targets the most socially underprivileged classes are advocated to promote safe motherhood and the objectives of antenatal care.

## Introduction

Prenatal care provided by skilled personnel has been found to ensure the health of pregnant mothers and improve pregnancy outcomes by identifying and promptly managing pregnancy-related complications [1-3]. The benefits of prenatal service utilization include provision of evidence-based clinical interventions, including prevention of mother-to-child transmission of HIV, maternal health education and counselling on birth-preparedness and complication-readiness. Specialized care can be quickly arranged when the need arises. Besides, women who attend prenatal care are more likely to have facility-based deliveries, and return for postnatal care [4, 5]. On the contrary, lack of antenatal care has been clearly linked with increased perinatal morbidity and mortality. While poor utilization of prenatal services is no longer an issue in most developed countries, low- and middle-income nations still grapple with the adverse pregnancy outcomes related to poor antenatal care [6-9]. Following large, randomized, multi-centre trials that identified evidence-based interventions, clinic-visit patterns that were beneficial and cost-effective to pregnant mothers, the World Health Organization (WHO) recommended four focused antenatal visits for all women [10]. This model has formed the basis for research designs, awareness campaigns and policy formulation in many nations since 2002. However, review of the benefits of the focused antenatal care model and the views of pregnant women led to the development of new guidelines in 2016. The latest model recommended a minimum of eight contacts to reduce pregnancy complications and perinatal mortality, and give women a positive pregnancy experience from antenatal care [11]. According to the Nigeria Demographic and Health Survey, among women who had a live birth in the five years preceding the survey, 61% received antenatal care from a skilled provider, while 51% of the pregnant women reportedly made at least four antenatal visits during the pregnancy [12]. This poor utilization of prenatal service occurs commonly in developing countries [13], but the predictors and barriers to accessing the minimum number of antenatal visits have not been fully explored in our setting. Nigeria is yet to operationalize the focused antenatal care model, nor the latest 2016 guidelines. Before the decision to fully transit from the traditional model to a new one is taken, it would be beneficial to evaluate the current practice in the light of the proposed models. This will serve as a template for policy makers involved in the development of public health strategies to increase antenatal coverage and improve prenatal service delivery.

## Methods

### Study site

The index study was conducted in Ekiti State University Teaching Hospital (EKSUTH), Ado-Ekiti, in Southern Nigeria, between April 2012 and March 2015. The hospital is a tertiary health facility which serves as the Teaching Hospital for the College of Medicine, Ekiti State University, Ado-Ekiti, and is also an obstetric referral centre serving the private, primary and secondary health institutions within Ekiti State and its neighbouring states. Its clientele is made up of a mixture of self-presenting, health-personnel-referred, and health-facility-transferred patients. It runs weekly antenatal clinics supervised by obstetricians, and all prenatal service delivery is undertaken by consultant-led teams.

### Study participants

The study participants were consecutive pregnant mothers who had their deliveries at the Teaching Hospital during the study period. Every week day, all obstetric cases managed in the preceding 24 hours in the Department of Obstetrics and Gynaecology are reviewed. Maternal socio-demographic and clinical characteristics were extracted into an electronic database using a comprehensive proforma with over 180 variables, including previous gynaecological and obstetric details, index pregnancy and its progress, parturition, puerperium, observed complications and perinatal outcomes. The data were obtained from antenatal progress records, registers in the materno-fetal medicine/antenatal ward, labour ward, maternity theatre, postnatal ward and complemented by entries from nurses´ sheets to ensure completeness. The extracted details were entered into the database by a trained research assistant employed full time for that purpose.

### Sample size

The size of the study population was purposively chosen to include women who delivered within the study duration. However, those who had incomplete data and women with severe medical illnesses necessitating frequent hospital visits were not included in the study. The institution´s Ethics and Research Committee gave approval for the study. For the purpose of this study, participants were regarded as unbooked for prenatal care if they had no antenatal care, were not cared for by a skilled provider, or referred from a traditional birth attendant following the occurrence of a complication during pregnancy or labour. The prenatal visits were contacts with the obstetricians and midwives according to the recommended schedule by the American College of Obstetricians and Gynaecologists [14] that did not result in delivery. Based on the number of prenatal visits, the women were divided into two operational groups: poor utilizers who had fewer than 4 visits and the good utilizers who had at least four. Gestational age at delivery was calculated from the number of weeks completed from the first day of the last menstrual period; if she was uncertain of her dates, this was extrapolated from ultrasonography. The birth weight was recorded at most six hours after birth to the nearest 100 grammes, while the Apgar scoring was done by the paediatrician or midwife present at the delivery in the first and fifth minutes of life. A baby had low birth weight if it weighed less than 2,500 grammes irrespective of the gestational age at delivery.

### Data analysis

The retrieved data were coded into, and analyzed using the Statistical Software for the Social Sciences (SPSS) package version 20. Frequency distribution and percentages were generated from the data. Pearson´s Chi-square test was used to explore the univariate association of the maternal and pregnancy characteristics with prenatal service utilization. Bivariate regression analysis was employed to test the strength of the association between maternal socio-demographic characteristics and pregnancy outcomes with prenatal service utilization, and the results were expressed as crude odds ratio with the corresponding 95% confidence interval. The variables that showed significant association with prenatal service utilization were included in multivariate logistic regression model to identify the independent predictors of poor prenatal service utilization. The results of the multivariate regression analyses were expressed as adjusted odds ratio at 95% confidence interval (C. I.), with level of significance set at p < 0.05.

## Results

Of the 2,139 parturients included in the study, the greater proportion, 1180 (55.2%), were at least 30 years old, married 2112 (98.7%), multiparous 1282 (59.9%), having a tertiary-level education 1646 (77%). They were mainly of Yoruba extraction, 1960 (91.6%), with other ethnic groups (such as Igala, Ebira, Isoko, etc.) making up 65 (3%) of the study population. Eighty percent (1713) of the women were employed, while 2020 (94.4%) had husbands who were in paid employment. Regarding their antenatal booking status, 624 (29.2%) of them were unbooked for antenatal care, while 159 (10.5%) had less than four antenatal visits before delivery (Table 1). The maternal characteristics and pregnancy outcome of unbooked women were compared with those of women who had less than four antenatal visits in Table 2. There was no statistically significant difference observed between the two groups of women. Women who had poor prenatal utilization were compared with adequate utilizers in Table 3. Poor utilizers were significantly more likely to be single mothers (30% versus 10.4%, p = 0.043), with unemployed husbands/partners (21.9% versus 10%, p = 0.002), but less likely to have labour induction (13.1% versus 3.2%, p < 0.001) compared with good utilizers. Also, the women with fewer than four antenatal visits had significantly more babies with low birth weight (18% versus 9.8%, p = 0.003), and 5-minute Apgar scores less than 7 (17.9% versus 10.1%, p = 0.023). Poor prenatal service utilization was predicted by having unemployed husbands/partners (adjusted odds ratio (AOR): 2.33; 95% C.I.: 1.24 - 4.38; p = 0.009). Also, not having induction of labour (AOR: 4.27; 95% C.I.: 2.38 - 7.64; p < 0.001) and having low-birth-weight babies (AOR: 1.66; 95% C.I.: 1.01 - 2.73; p = 0.045) were independently associated with poor prenatal service utilization (Table 4).

**Table 1:** Maternal socio-demographic characteristics, n = 2,139

Characteristics	Categories	Frequency	Percentage
**Age (years)**	< 30	959	44.8
	≥ 30	1180	55.2
**Parity**	Nulliparous	857	40.1
	Multiparous	1282	59.9
**Marital status**	Single	27	1.3
	Married	2112	98.7
**Maternal education**	No formal	10	0.5
	Primary	48	2.2
	Secondary	435	20.3
	Tertiary	1646	77
**Employment (wife)**	Unemployed	426	19.9
	Employed	1713	80.1
**Employment (husband)**	Unemployed	119	5.6
	Employed	2020	94.4
**Ethnicity**	Yoruba	1960	91.6
	Igbo	110	5.1
	Hausa	4	0.2
	Others	65	3
**Prenatal booking status**	Booked	1515	70.8
	Unbooked	624	29.2
**Prenatal service utilization (n = 1515)**	Poor (< 4 visits)	159	10.5
	Good (≥ 4 visits)	1356	89.5

Categorization of the patients into ‘good’ and ‘poor’ service utilizers was done only for clients who received prenatal care in the study location.

**Table 2:** Maternal socio-demographic characteristics and pregnancy outcome of women without prenatal care versus women with poor prenatal service utilization

Characteristics	Categories	Frequency of ANC visits		χ2	p value
		None	< 4		
		n (%)	n (%)		
**Age (years)**	< 30	298 (47.8)	78 (49.1)	0.086	0.770
	≥ 30	326 (52.2)	81 (50.9)		
**Parity**	Nulliparous	251 (40.2)	63 (39.6)	0.019	0.890
	Multiparous	373 (59.8)	96 (60.4)		
**Marital status**	Single	17 (2.7)	3 (1.9)	0.357	0.550
	Married	607 (97.3)	156 (98.1)		
**Employment (wife)**	Unemployed	143 (22.9)	37 (23.3)	0.009	0.925
	Employed	481 (77.1)	122 (76.7)		
**Employment (husband)**	Unemployed	55 (8.8)	14 (8.8)	0.000	0.997
	Employed	569 (91.2)	145 (91.2)		
**Maternal education**	No formal	3 (0.5)	1 (0.6)	1.197	0.754
	Primary	15 (2.4)	2 (1.3)		
	Secondary	162 (26)	38 (23.9)		
	Tertiary	444 (71.2)	118 (74.2)		
**Ethnicity**	Yoruba	570 (91.3)	148 (93.1)	0.628	0.730
	Igbo	31 (5)	7 (4.4)		
	Hausa	0 (0)	0 (0)		
	Others	23 (3.7)	4 (2.5)		
**Previous stillbirth**	Yes	25 (4)	9 (5.7)	0.835	0.361
	No	599 (96)	150 (94.3)		
**Fetal sex**	Male	336 (53.8)	84 (52.8)	0.053	0.819
	Female	288 (46.2)	75 (47.2)		
**Labour induction**	No	560 (89.7)	146 (91.8)	0.618	0.432
	Yes	64 (10.3)	13 (8.2)		
**Gestational Age at birth**	< 37 weeks	139 (22.3)	35 (22)	0.005	0.943
	≥ 37 weeks	485 (77.7)	124 (78)		
**Mode of delivery**	Vaginal	367 (58.8)	104 (65.4)	2.299	0.129
	Caesarean	257 (41.2)	55 (34.6)		
**Birth weight (grammes)**	< 2,500	89 (14.3)	29 (15.1)	0.071	0.790
	≥ 2,500	535 (85.7)	135 (84.9)		
**Stillbirth**	No	594 (95.2)	152 (95.6)	0.046	0.830
	Yes	30 (4.8)	7 (4.4)		
**^1^NICU admission**	No	538 (86.2)	142 (89.3)	1.059	0.303
Yes	86 (13.8)	17 (10.7)		
**5-minute APGAR**	< 7	53 (8.5)	15 (9.4)	0.141	0.707
	≥ 7	571 (91.5)	144 (90.6)		

1 NICU = Neonatal Intensive Care Unit

Poor utilizers had less than 4 visits at the study location; women categorized as ‘none’ utilizers were not booked for prenatal care in the study location.

**Table 3:** Maternal socio-demographic characteristics and pregnancy outcome of women with poor prenatal service utilization versus women with good prenatal service utilization

Characteristics	Prenatal service utilization		χ2	p value
	Poor (< 4 visits)	Good (≥ 4 visits)		
	n (%)	n (%)		
**Age (years)**				
< 30	78 (11.8)	583 (88.2)	2.127	0.145
≥ 30	81 (9.5)	773 (90.5)		
**Parity**				
Nulliparous	63 (10.4)	543 (89.6)	0.011	0.918
Multiparous	96 (10.6)	813 (89.4)		
**Marital status**				
Single	3 (30)	7 (70)	4.077	0.043*
Married	156 (10.4)	1349 (89.6)		
**Ethnicity**				
Yoruba	148 (10.6)	1242 (89.4)	0.770	0.857
Igbo	7 (8.9)	72 (91.1)		
Hausa	0 (0)	4 (100)		
Others	4 (9.5)	38 (90.5)		
**Maternal education**				
No formal	1 (14.3)	6 (85.7)	4.794	0.188
Primary	2 (6.1)	31 (93.9)		
Secondary	38 (13.9)	235 (86.1)		
Tertiary	118 (9.8)	1084 (90.2)		
**Employment (wife)**				
Unemployed	37 (13.1)	246 (86.9)	2.464	0.116
Employed	122 (9.9)	1110 (90.1)		
**Employment (husband)**				
Unemployed	14 (21.9)	50 (78.1)	9.212	0.002*
Employed	145 (10)	1306 (90)		
**Previous stillbirth**				
Yes	9 (12.3)	64 (87.7)	0.275	0.600
No	150 (10.4)	1292 (89.6)		
**Labour induction**				
No	146 (13.1)	969 (86.9)	30.370	<0.001*
Yes	13 (3.2)	387 (96.8)		
**Gestational age at birth**				
< 37 weeks	35 (13.7)	220 (86.3)	3.406	0.065
≥ 37 weeks	124 (9.8)	1136 (90.2)		
**Mode of delivery**				
Vaginal	104 (10.3)	908 (89.7)	0.155	0.694
Caesarean	55 (10.9)	448 (89.1)		
**Fetal sex**				
Male	84 (10.4)	720 (89.6)	0.004	0.949
Female	75 (10.5)	636 (89.5)		
**5-minute APGAR**				
< 7	15 (17.9)	69 (82.1)	5.131	0.023*
≥ 7	144 (10.1)	1287 (89.9)		
**Birth weight (grammes)**				
< 2,500	24 (18)	109 (82)	8.848	0.003*
≥ 2,500	135 (9.8)	1247 (90.2)		
**Stillbirth**				
No	152 (10.3)	1325 (89.7)	2.607	0.106
Yes	7 (18.4)	31 (81.6)		
**^1^NICU admission**				
No	142 (10.5)	1211 (89.5)	0.000	1.000
Yes	17 (10.5)	145 (89.5)		

1 NICU = Neonatal Intensive Care Unit;

* significant at p < 0.05

Unlike in Table 2, all the women compared in this table had prenatal care at the study location. The adequacy of their visits was the yardstick.

**Table 4:** Logistic regression analyses of predictors of poor prenatal service utilization

Characteristics	Prenatal service utilization		Crude odds ratio (95% Confidence Interval)	p-value	Adjusted odds ratio (95% Confidence Interval)	p-value
	Poor (< 4 visits)	Good (≥ 4 visits)				
	n (%)	n (%)				
**Age (years)**						
< 30	78 (11.8)	583 (88.2)	1.00			
≥ 30	81 (9.5)	773 (90.5)	1.27 (0.92 - 1.77)	0.145		
**Parity**						
Nulliparous	63 (10.4)	543 (89.6)	1.00			
Multiparous	96 (10.6)	813 (89.4)	0.98 (0.70 – 1.38)	0.918		
**Marital status**						
Single	3 (30)	7 (70)	1.00			
Married	156 (10.4)	1349 (89.6)	3.71 (0.95 – 14.48)	0.060		
**Ethnicity**						
Yoruba	148 (10.6)	1242 (89.4)	0.88 (0.31 – 2.51)	0.816		
Igbo	7 (8.9)	72 (91.1)	1.08 (0.30 – 3.93)	0.904		
Hausa	0 (0)	4 (100)	1.70E8 (0.00 - ∞)	0.999		
Others	4 (9.5)	38 (90.5)	1.00			
**Maternal education**						
No formal	1 (14.3)	6 (85.7)	1.00			
Primary	2 (6.1)	31 (93.9)	2.58 (0.20 – 33.24)	0.467		
Secondary	38 (13.9)	235 (86.1)	1.03 (0.12-8.80)	0.978		
Tertiary	118 (9.8)	1084 (90.2)	1.53 (0.18 – 12.83)	0.694		
**Employment (wife)**						
Unemployed	37 (13.1)	246 (86.9)	1.00			
Employed	122 (9.9)	1110 (90.1)	1.37 (0.92 – 2.03)	0.118		
**Employment (husband)**						
Unemployed	14 (21.9)	50 (78.1)	1.00		1.00	
Employed	145 (10)	1306 (90)	2.52 (1.36 – 4.67)	0.003*	2.33 (1.24 – 4.38)	0.009*
**Previous stillbirth**						
Yes	9 (12.3)	64 (87.7)	1.00			
No	150 (10.4)	1292 (89.6)	1.21 (0.59 – 2.48)	0.601		
**Labour induction**						
No	146 (13.1)	969 (86.9)	1.00		1.00	
Yes	13 (3.2)	387 (96.8)	4.49 (2.51 – 8.01)	<0.001*	4.27 (2.38 – 7.64)	<0.001*
**Gestational age at birth**						
< 37 weeks	35 (13.7)	220 (86.3)	1.00			
≥ 37 weeks	124 (9.8)	1136 (90.2)	1.46 (0.98 – 2.18)	0.066		
**Mode of delivery**						
Vaginal	104 (10.3)	908 (89.7)	1.00			
Caesarean	55 (10.9)	448 (89.1)	0.93 (0.66 – 1.32)	0.694		
**Fetal sex**						
Male	84 (10.4)	720 (89.6)	1.00			
Female	75 (10.5)	636 (89.5)	0.99 (0.71 – 1.38)	0.949		
**5-minute APGAR**						
< 7	15 (17.9)	69 (82.1)	1.00		1.00	
≥ 7	144 (10.1)	1287 (89.9)	1.94 (1.08 – 3.49)	0.026*	1.57 (0.86 – 2.89)	0.143
**Birth weight (grammes)**						
< 2,500	24 (18)	109 (82)	1.00		1.00	
≥ 2,500	135 (9.8)	1247 (90.2)	2.03 (1.26 – 3.28)	0.003*	1.66 (1.01 – 2.73)	0.045*
**Stillbirth**						
No	152 (10.3)	1325 (89.7)	1.00			
Yes	7 (18.4)	31 (81.6)	0.51 (0.22 – 1.17)	0.113		
**^1^NICU admission**						
No	142 (10.5)	1211 (89.5)	1.00			
Yes	17 (10.5)	145 (89.5)	1.00 (0.59 – 1.70)	1.000		

1 NICU = Neonatal Intensive Care Unit;

* significant at p < 0.05

Predictors and pregnancy outcomes of good and poor utilizers were determined in this table.

## Discussion

Our study did not just seek to compare women who had antenatal care at EKSUTH with those who did not, because that would underestimate the beneficial value of prenatal care. Instead, we sought to explore the potential advantages of adequate antenatal visits/service utilization over poor utilization with respect to pregnancy outcomes.

### Statement of principal findings

Interestingly, we found that women who did not utilize prenatal services by a skilled provider were comparable in maternal characteristics and pregnancy outcomes with those who were poor utilizers. Women´s decision to fully maximize the potential benefits of prenatal services in low-income settings is modified by certain factors, including ease of accessing the facility, their perception of the quality of service provided, socio-economic status, level of education, previous pregnancy outcomes and cultural beliefs [7, 15, 16]. Eliminating these barriers would increase maternal prenatal service utilization. Single/unmarried mothers were more likely to utilize prenatal services poorly. This finding, which has been documented by other authors [17], can be due to lack of social support, teenage pregnancies, unwanted pregnancies and fear of stigmatization. We found that women with unemployed spouses were less likely to fully utilize antenatal care packages. In our largely paternalistic setting, the socio-economic status of women with unemployed husbands may likely be low, which may negatively impact on their attendance at antenatal clinics where they need to pay out-of-pocket [18-20]. Our study also revealed that, more women with poor antenatal attendance had babies with 5-minute Apgar scores below 7. The association between lack of antenatal care and perinatal morbidity and mortality has been documented by studies from various countries [6]. This could be the result of an interplay of poor antenatal attendance with social factors like poor health-seeking behaviours of the mothers. Besides, this category of women might belong to a socially underprivileged group (such as low family income) with increased risk of adverse perinatal outcome [6]. However, having unemployed partners was the only one of these three variables that was associated with poor utilization after controlling for other confounders. The index study revealed that poor utilizers of prenatal care were significantly less likely to have labour induction. Several Nigerian studies have documented that the commonest indication for labour induction is post-term pregnancies [21-23]. This may suggest that more women who utilized prenatal care poorly did not exceed their expected dates of delivery, a likely scenario corroborated by the finding that more low birth weight babies were delivered by poor utilizers of prenatal care. There are a number of perspectives to this latter observation. On the one hand, risk factors for intrauterine fetal restriction might not be identified early in women with less than four antenatal visits, with delayed commencement of management options and resultant low birth weight babies. On the other hand, they may present with complications in pregnancy necessitating earlier deliveries. Considering the link between poor perinatal utilization, low birth weight and increasing perinatal/childhood challenges [6, 24-27], efforts at scaling up awareness campaigns on maximizing the benefits of prenatal care and increasing the content quality of antenatal visits to give women a positive pregnancy experience are advocated.

### Strengths and weaknesses of the study

The results of this facility-based study may not be an exact reflection of the situation in the community. However, since the study setting receives referrals from the primary and secondary health facilities that are closer to the community, the findings may be extrapolated to represent the local population. Also, although the data did not include evaluation of the content of the antenatal visits, this survey has provided salient data that can serve as a template for further studies on the subject.

### Meaning of the study

Mothers who had no or less-than-adequate antenatal care have unfavourable perinatal outcomes. Identifying predictors of poor utilization could guide public health interventions aimed at increasing uptake of antenatal care.

### Unanswered questions and future research

It is not completely certain if provision of universal access to antenatal care will lead to total acceptance and uptake of prenatal service in our setting. The influence of socio-cultural modifiers of health-seeking behaviour as it relates to prenatal service uptake needs to be explored.

## Conclusion

The socio-demographic characteristics and pregnancy outcomes of mothers who had no skilled prenatal care provider did not differ significantly from those of mothers who utilized prenatal services poorly. Furthermore, women who had less than four prenatal visits were more likely to be single, with unemployed partners, and have babies with low birth weight and poor 5-minute Apgar scores. Efforts devoted to identifying women who are likely to be non- and poor-utilizers of prenatal care are recommended. Eliminating barriers to accessing prenatal services, and implementing a National Health Insurance package that also targets the most socially underprivileged classes are advocated.

### What is known about this topic

- Utilizing antenatal care adequately improves pregnancy outcomes;
- Developing nations still grapple with the unpleasant outcomes of poor antenatal care.

### What this study adds

- Having no care is similar to poor antenatal care in terms of pregnancy outcomes;
- Indices of low socioeconomic status are predictors of poor antenatal utilization;
- Poor perinatal outcomes are linked with poor antenatal care; therefore, Nigeria should adopt a model that will ensure improved materno-fetal well-being via increased clinic visits.
